# Tumor cell-produced matrix metalloproteinase 9 (MMP-9) drives malignant progression and metastasis of basal-like triple negative breast cancer

**DOI:** 10.18632/oncotarget.1932

**Published:** 2014-05-01

**Authors:** Christine Mehner, Alexandra Hockla, Erin Miller, Sophia Ran, Derek C. Radisky, Evette S. Radisky

**Affiliations:** ^1^ Department of Cancer Biology, Mayo Clinic, Jacksonville, FL; ^2^ Department of Medical Microbiology, Immunology and Cell Biology, Southern Illinois University School of Medicine, Springfield, IL

**Keywords:** triple negative breast cancer, basal-like breast cancer, matrix metalloproteinases, invasion, metastasis, mouse orthotopic tumor models, bioluminescence imaging

## Abstract

Matrix metalloproteinases (MMPs) have been implicated in diverse roles in breast cancer development and progression. While many of the different MMPs expressed in breast cancer are produced by stromal cells MMP-9 is produced mainly by the tumor cells themselves. To date, the functional role of tumor cell-produced MMP-9 has remained unclear. Here, we show that human breast cancer cell-produced MMP-9 is specifically required for invasion in cell culture and for pulmonary metastasis in a mouse orthotopic model of basal-like breast cancer. We also find that tumor cell-produced MMP-9 promotes tumor vascularization with only modest impact on primary tumor growth, and that silencing of MMP-9 expression in tumor cells leads to an altered transcriptional program consistent with reversion to a less malignant phenotype. MMP-9 is most highly expressed in human basal-like and triple negative tumors, where our data suggest that it contributes to metastatic progression. Our results suggest that MMP9 may offer a target for anti-metastatic therapies for basal-like triple negative breast cancers, a poor prognosis subtype with few available molecularly targeted therapeutic options.

## INTRODUCTION

Breast cancer is the most frequently diagnosed cancer in women in the United States, and accounts for the second highest number of cancer deaths [1]. Breast cancers can be classified into subtypes based on expression of histopathological markers or on gene expression profiles; using either approach, different subtypes have markedly different prognostic outlooks [2-5]. Major differences in survival among breast cancer subtypes derive in part from the availability of appropriate molecularly targeted adjuvant therapies and in part from the intrinsic aggressiveness of the cancer subtypes. While the introduction of tamoxifen and trastuzumab have improved outcomes for patients with hormone receptor-positive and HER2-positive cancers, respectively [6, 7], no equivalent targeted agents have yet been identified for breast cancers of the “triple negative” histological subtype (negative for estrogen receptor (ER), progesterone receptor (PR), and HER2) and the molecularly defined basal-like subtype [8, 9]. Furthermore, triple negative or basal-like cancers are more often highly invasive, spreading to lymph nodes while primary tumors are still small, and leading to early relapse with distant metastasis [9-11]. Outlooks for these high risk groups can be improved by unraveling the mechanisms that enable uncontrolled cancer spread, and by identifying new points of therapeutic intervention particularly for those breast cancer subtypes that are refractory to currently available molecularly targeted therapies.

The matrix metalloproteinases (MMPs) are a family of zinc-dependent endopeptidases with important functions in extracellular matrix remodeling during development and in inflammation and wound repair processes [12, 13]. As regulators of the tumor microenvironment, they are also important contributors to cancer initiation, development, and progression via multiple mechanisms [14, 15]. Of particular relevance to metastasis, MMPs degrade basement membranes and expose cryptic peptide epitopes in the extracellular matrix, stimulating cellular invasion [14, 15]. They also directly modify integrins and other cancer cell adhesion molecules, and proteolytically activate powerful cytokines such as TGF-β, leading to induction of epithelial-mesenchymal transition, a comprehensive phenotypic alteration characterized by enhanced cell motility [14-16]. MMPs at the primary tumor site can also release soluble factors into circulation that can help to establish a metastatic niche in distant organs, aiding subsequent colonization by tumor cells [14]. Interestingly, MMPs are produced both by tumor cells themselves and by complicit stromal cells in the tumor microenvironment, in response to reciprocal paracrine stimulatory interactions [17].

Matrix metalloproteinase 9 (MMP9), also known as gelatinase B, is an MMP that is strongly associated with aggressive and metastatic breast cancer. It is one of 70 genes in the Rosetta poor prognosis signature for breast cancer patients [3], and also correlates with poor prognosis in other breast cancer DNA microarray datasets [18, 19]. The relevant source of MMP9 as a poor prognosis marker in human cancer remains unclear. Experimental metastasis studies in mouse models in which lung carcinoma or melanoma cells were injected into MMP9-deficient mice showed that host MMP9 promotes metastatic colonization of the lung [20, 21]. Metastatic colonization was enhanced in MMP9-knockout mice implanted with MMP9-expressing bone marrow [20], and MMP9 also promotes lung metastasis in the MMTV-PyVT multistage mammary tumor model, potentially deriving from the pro-angiogenic contribution of neutrophil MMP9 in the lungs [22]. Such studies support the concept that MMP9 can promote metastasis when expressed by stromal cells, at least in mouse models. However, several large studies examining MMP9 expression in human tumors by immunohistochemistry have demonstrated that MMP9 is most widely produced by tumor cells, with lower incidence of expression in fibroblasts and immune cells [23-26]. While MMP9 expression in stromal cells has important prognostic implications in human breast cancer [24, 25], the significance of the more substantial expression of MMP9 expression by tumor cells has not been clarified. While in some studies high expression of MMP9 by tumor cells was associated with higher rates of lymph node metastasis [27, 28], distant metastasis [25], and poorer relapse-free survival [23, 25], other studies have reported associations of tumor cell MMP9 expression with better overall survival or recurrence-free survival [24, 26].

Here, by manipulating tumor cell MMP9 expression in breast cancer cell lines and in an orthotopic mouse model of metastatic human breast cancer, we dissect the functional significance of tumor cell-produced MMP9 for invasion and metastasis. Our results specifically implicate tumor cell-produced MMP9 in invasion and metastatic progression in models of basal-like triple negative breast cancer, and suggest MMP9 as a potential therapeutic target to combat metastasis in this poor prognosis breast cancer subtype for which few current molecularly targeted therapies are effective.

## RESULTS

### Tumor cell-produced MMP9 expression drives invasiveness of basal-like triple negative breast cancer cell lines

**Figure 1:**
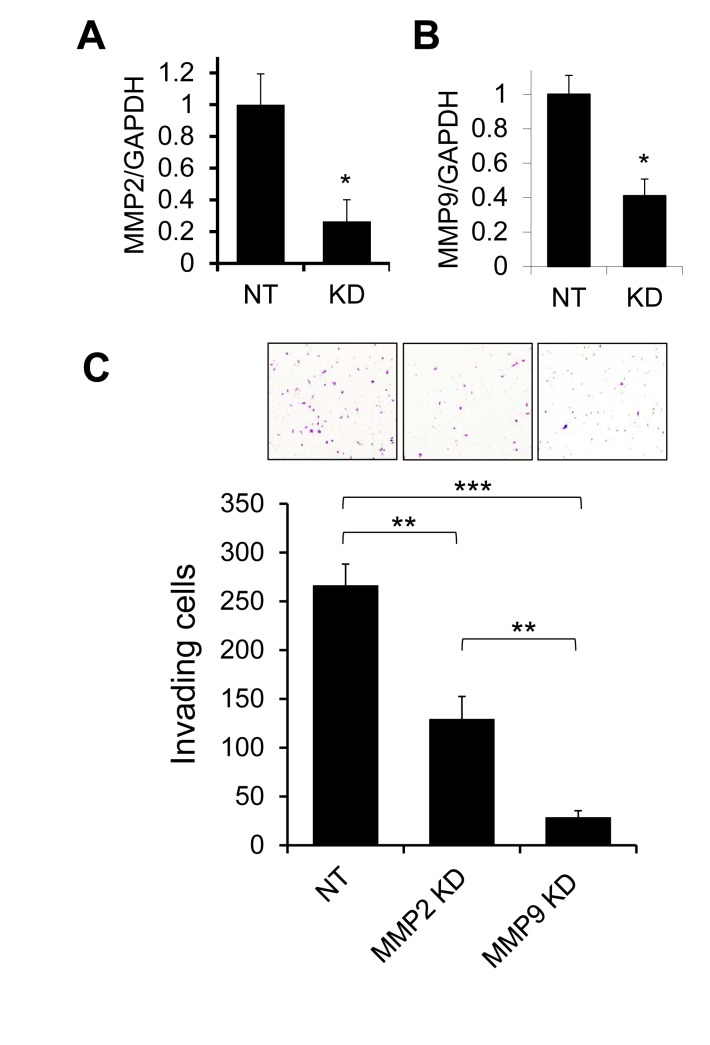
Effects of MMP2 and MMP9 silencing on invasion of MDA-MB-231 cells (A,B) Transduction of MDA-MB-231 cells with lentiviral shRNA constructs specifically targeting MMP2 (A) or MMP9 (B) resulted in significant suppression of expression of the targeted transcripts as assessed by qRT/PCR, by comparison with control cells transduced with a nontarget construct recognizing no human genes (NT). (C) In Matrigel transwell invasion assays, MDA-MB-231 cells with knockdown of MMP2 or MMP9 showed significantly reduced invasion relative to control cells (NT). Graph shows mean and SEM for quadruplicate biological replicates. Representative fields from invasion filters are shown above graphical results. *, p<0.05; **, p<0.01; ***, p<0.0001 (unpaired *t* test).

We have previously found that tumors derived from MDA-MB-231 human breast cancer cells orthotopically implanted in mice show evidence of gelatinase activity, and that treatment of MDA-MB-231 cells with tissue inhibitor of metalloproteinases-1 (TIMP-1) can reduce cellular invasiveness [29]. Because transcripts for gelatinases MMP2 and MMP9 are both expressed at low but detectable levels in MDA-MB-231 cells, we evaluated the impact of individually silencing MMP2 or MMP9 expression using targeted lentiviral shRNA constructs, to identify the specific gelatinase(s) responsible for promoting invasion (Fig. 1). Both MMP2 and MMP9 expression could be effectively diminished by shRNA knockdown (Fig. 1A,B). MMP2 knockdown reduced cellular invasiveness in Matrigel transwell assays by about 50%, while MMP9 knockdown had a much more substantial effect, reducing invasion by 90% (Fig. 1C), suggesting MMP9 as a major effector of invasive behavior in MDA-MB-231 cells.

**Figure 2:**
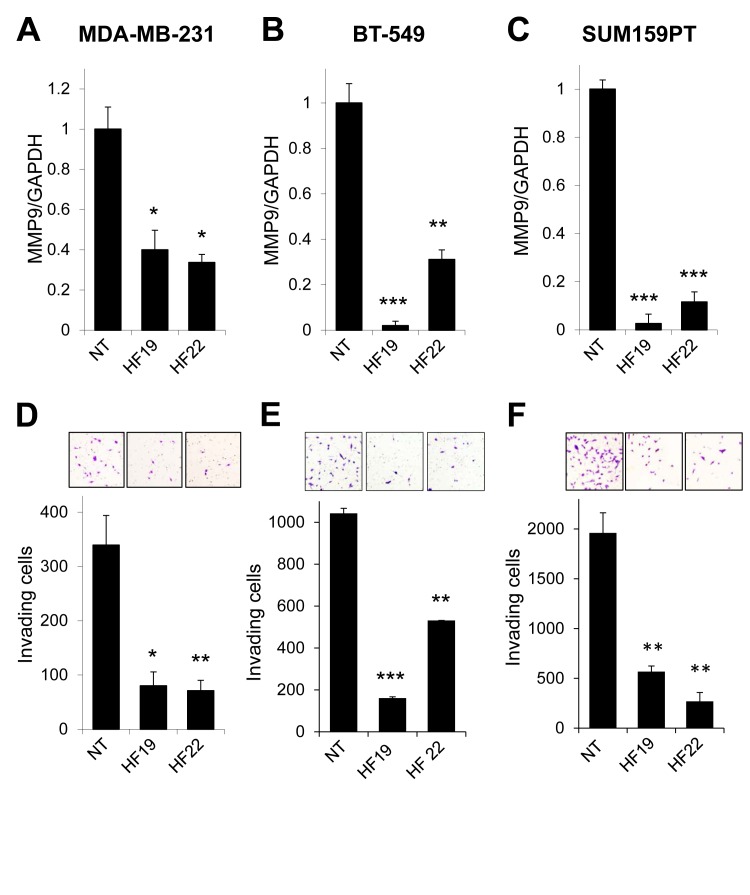
MMP9 silencing inhibits invasion in models of basal-like, triple negative breast cancer (A-C) Transduction of (A) MDA-MB-231, (B) BT-549, or (C) SUM159PT cells with two different lentiviral shRNA constructs specifically targeting MMP9 (designated HF19 and HF22) suppressed MMP9 transcript levels in comparison to control cells transduced with a nontarget control construct (NT). (D-F) Knockdown of MMP9 in (D) MDA-MB-231, (E) BT-549, or (F) SUM159PT cells by shRNAs HF19 and HF22 suppressed cellular invasion in Matrigel transwell assays. Graphs shows mean and SEM for triplicate biological replicates; representative fields from invasion filters are shown above graphical results. *, p<0.05; **, p<0.01; ***, p<0.0001 (unpaired t-test).

To extend these initial observations, we carried out MMP9 silencing experiments using multiple MMP9-targeted lentiviral shRNA constructs and additional cell lines. MDA-MB-231 cells are triple negative, and represent an accepted model of the basal-like breast cancer subtype [30, 31]. Triple negative breast cancers are often aggressive and have a high risk of early metastatic relapse [8, 9]. Hypothesizing that MMP9 may act as a general driver of the invasive/metastatic propensities of triple negative breast cancers, we evaluated the impact of MMP9 knockdown on basal-like, triple negative breast cancer cell lines BT-549 and SUM159PT. Knockdown of MMP9 expression in MDA-MB-231 cells (Fig 2A), BT-549 cells (Fig. 2B), and SUM159PT cells (Fig. 2C) led to consistent suppression of invasiveness in Matrigel transwell assays (Fig. 2D,E, F, respectively).

### Tumor cell-produced MMP9 is essential for metastasis in an orthotopic xenograft model of basal-like triple negative breast cancer

**Figure 3:**
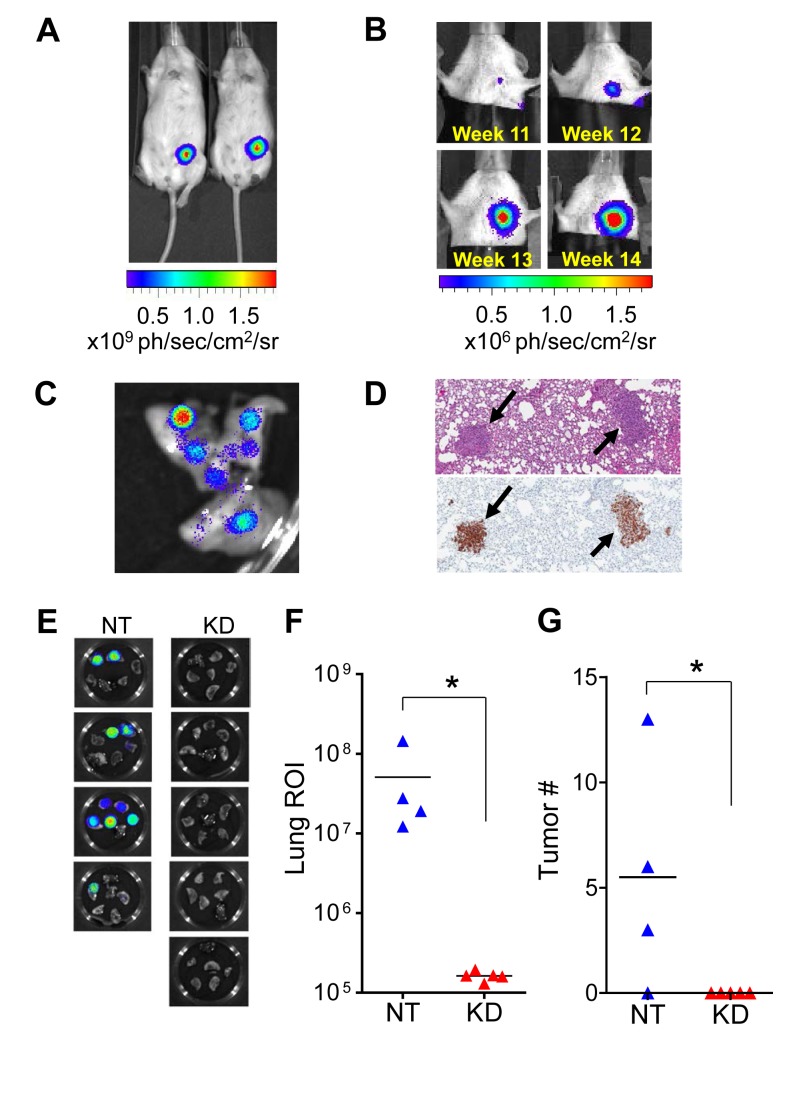
MMP9 silencing blocks metastasis to lung in mouse orthotopic model (A) When MDA-MB-231-luc2 cells expressing firefly luciferase were implanted orthotopically into the mammary fat pad of Nod/Scid mice (1×10^5^ cells), tumor growth could be followed *in vivo* by bioluminescence imaging. (B) Spontaneous metastasis of MDA-MB-231-luc2 orthotopic tumors to the lungs was detected within 11 weeks using *in vivo* bioluminescence imaging. (C) Tumor-bearing mice were injected with luciferin shortly before euthanization to enable detection of metastases (as in the lung lobes shown here) and quantification of tumor burden by *ex vivo* imaging. (D) Metastases in the lungs (indicated by black arrows) were confirmed after formalin fixation and paraffin embedding by hematoxylin and eosin staining (top) and by immunohistochemical staining for human cytokeratins (bottom). (E) Mice implanted with 1×10^5^ MDA-MB-231-luc2 cells in which MMP9 was knocked down with lentiviral shRNA (KD; n=5) and euthanized after 11 weeks showed no evidence of pulmonary metastasis by *ex vivo* bioluminescence imaging of the excised lungs, while all mice implanted with 1×10^5^ control cells transduced with a nontarget lentivirus (NT, n=4) revealed bioluminescent signal diagnostic of metastasis. This difference was highly significant (p=0.0079; Fisher exact test). (F) Quantification of metastatic tumor burden in the lungs by bioluminescence flux showed significant differences between the two groups (p=0.0159, Mann-Whitney test). (G) Metastatic tumor number was assessed by counting individual metastatic lesions in a single section through all lung lobes of each mouse; this result also confirmed significant difference between the groups (p=0.04162; Wilcoxon Rank Sum test with continuity correction).

To evaluate the role of tumor cell-produced MMP9 in tumor progression and metastasis *in vivo*, we implemented an orthotopic xenograft model in which MDA-MB-231 cells stably expressing firefly luciferase (MDA-MB-231-luc2, Caliper Life Sciences) were implanted into the mammary fat pad of immunocompromised Nod/Scid mice. In this model, primary tumor growth could be followed *in vivo* by bioluminescence imaging (Fig. 3A), which detected evidence of metastasis by 11 weeks after tumor cell implantation (Fig. 3B). *Ex vivo* bioluminescence imaging (Fig. 3C) and immunohistochemistry (Fig. 3D) confirmed the presence of pulmonary metastases in mice for which metastasis was detected *in vivo*.

Mice were implanted with 1×10^5^ MDA-MB-231-luc2 cells stably transfected either with a nontarget control virus (NT) or with an MMP9-targeted lentiviral shRNA construct (KD). Mice were monitored regularly by *in vivo* bioluminescence imaging and euthanized at 11 weeks post-implantation. *Ex vivo* imaging showed that all of the control mice had evidence of pulmonary metastasis, whereas none of the mice bearing tumors in which MMP9 was silenced showed any bioluminescence signal in the lungs (Fig. 3E); this difference in outcomes was highly significant (p=0.0079; Fisher exact test). The extent of pulmonary metastasis in each mouse was quantified by bioluminescence flux (Fig. 3F) and by histopathological and histochemical analysis of a single section through all lung lobes (Fig. 3G), each of which likewise showed significant differences between groups.

### Tumor cell-produced MMP9 promotes vessel formation in an orthotopic xenograft model of basal-like triple negative breast cancer

**Figure 4:**
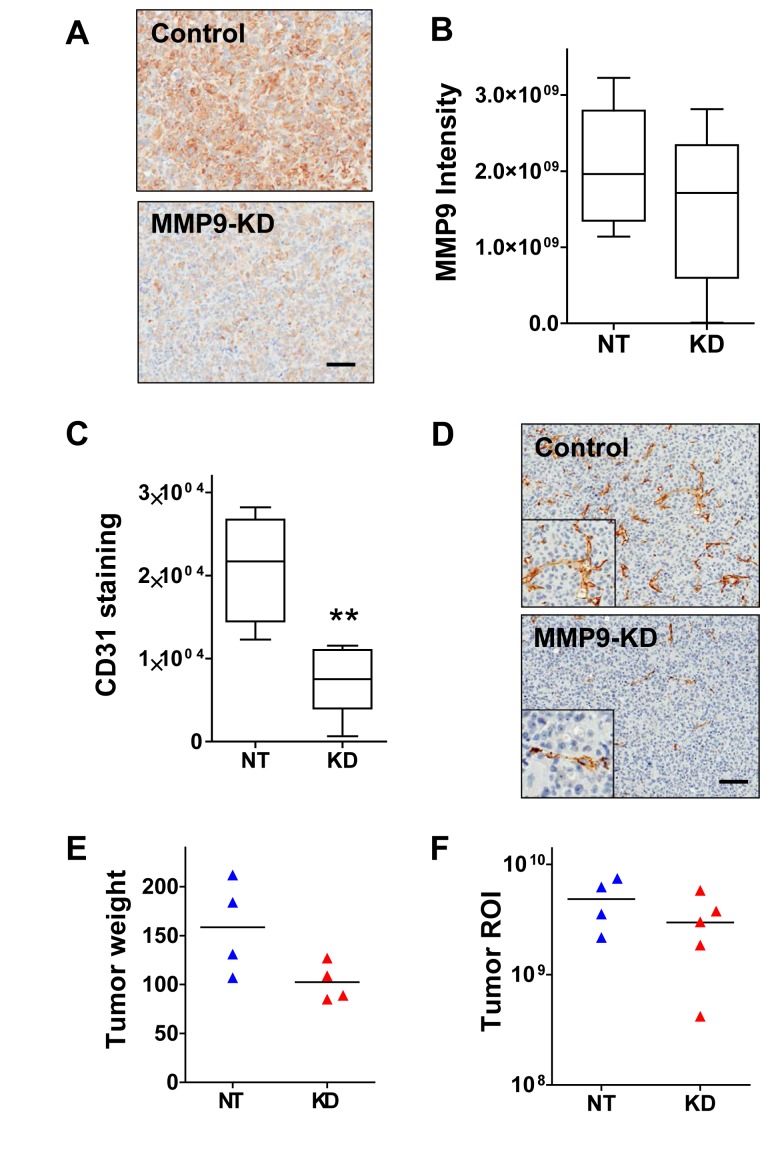
MMP9 silencing inhibits vessel formation and modestly suppresses tumor growth (A) Representative images of a control tumor (top) and an MMP9-KD tumor (bottom) stained for MMP9 illustrate typical intensity and distribution of the MMP9 staining in primary tumors. (B) Tumor sections stained for MMP9 were scanned and subjected to image analysis to quantify overall stain intensity. Tumors derived from MMP9-KD cells showed reduced intensity compared to control tumors; however, the differenced did not reach statistical significance. (C) Primary tumor sections were stained for CD31 and overall staining intensity was quantified by image analysis. Tumors established from MMP9-KD cells showed significantly reduced staining for CD31 relative to tumors established from NT control cells (p=0.0074, unpaired t-test). (D) Representative image of a control tumor section stained for CD31 (top) shows typical staining of endothelial cells forming well-developed vessels. Image of an MMP9-KD tumor section stained for CD31 (bottom) shows reduced number of cells positive for CD31 and disrupted vascular morphology. (E) Resected primary tumors from the MMP9 knockdown group weighed slightly less than tumors from the control mice, although the difference did not reach statistical significance. (F) *Ex vivo* imaging of resected MMP9 KD primary tumors similarly showed slightly reduced luminescence flux relative to controls (not significant).

Excised primary tumors from the above experiment were also examined for effects of MMP9 suppression. Tumor sections stained for MMP9 protein expression showed a trend of lower overall stain intensity in the MMP9 KD group, although there was considerable variation within each group (Fig. 4A,B), suggesting that the knockdown was not uniformly maintained throughout the latter part of the 11 week experimental time course.

MMP9 has been implicated previously as a critical mediator in the processes of tumor angiogenesis and vasculogenesis [32, 33], and so we also assessed the extent of tumor vascularization by staining the tumors for endothelial cell marker CD31. The tumors of the MMP9 KD group had significantly reduced staining compared to the control group (Fig. 4C; p=0.0159, Mann Whitney test), as a result of fewer CD31 positive cells (Fig. 4D). Furthermore, the CD31 staining pattern showed that the vascular morphology also differed between the two groups; whereas control tumors possessed properly formed blood vessels with structured lumina, the MMP9 KD tumor vasculature was more disorganized and often lacked lumina (Fig. 4D). Surprisingly, the reduction in blood vessel density did not appear to correlate with a reduction in tumor growth, as the average tumor weight (Fig. 4E) and *ex vivo* bioluminescence signal (Fig. 4F) were only slightly lower in the group with tumor cell MMP9 KD (not significant).

### MMP9 is associated with a tumorigenic expression profile in MDA-MB-231 cells

**Figure 5:**
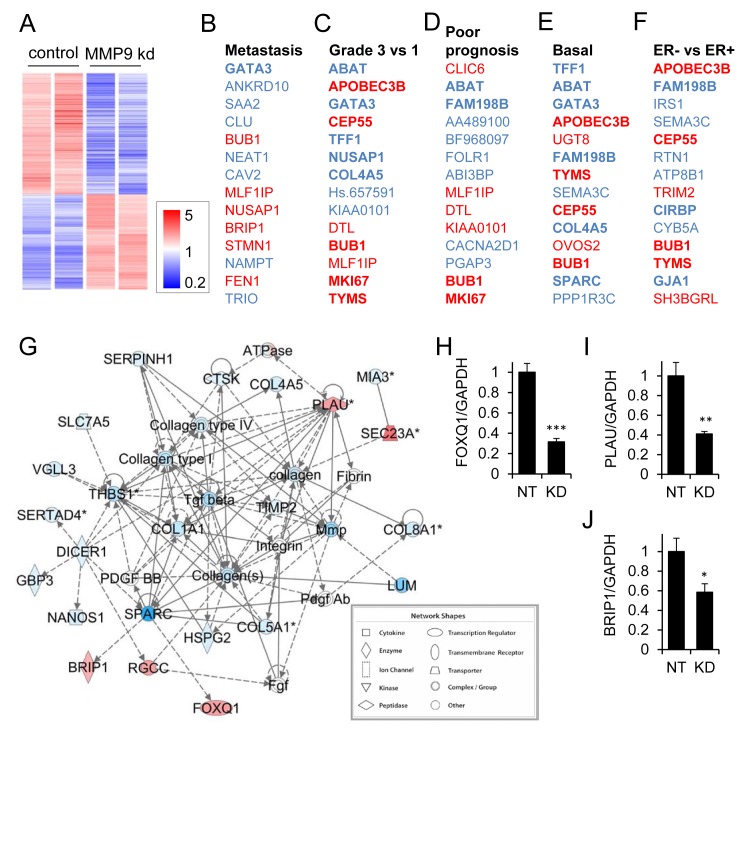
MMP9 is associated with a tumorigenic expression profile in MDA-MB-231 cells (A) Heat map of 1423 transcripts were differentially regulated in MMP9 KD vs NT cells (p<0.05, FC>2). (B-F) Top 14 coregulated transcripts in MMP9 NT vs KD cells and published datasets comparing metastatic breast cancer vs nonmetastatic breast cancer (B), grade 3 vs 1 breast cancers (C), poor prognosis vs better prognosis breast cancers (D), basal intrinsic subtype vs other subtypes (E), and ER- vs ER+ breast cancers (F); upregulated transcripts are red; downregulated transcripts are blue. (G) Ingenuity Pathway Analysis (IPA) network of genes differentially regulated in MMP9 NT vs MMP9 KD cells (intensity of red indicates degree of increased expression in MMP9 NT vs MMP9 KD). Direct interactions are indicated by solid lines, indirect by dashed lines. (H-J) Transcript levels of FOXQ1 (H), PLAU (I), and BRIP1 (J) for nontarget control (NT) and MMP9 knockdown (KD) MDA-MB-231 cells were assessed by qRT/PCR and normalized *vs.* GAPDH. *, p<0.05; **, p<0.01; ***, p<0.0001 (unpaired *t* test).

To investigate how the tumor cell-produced MMP9 drives the invasive/metastatic phenotype of triple negative/basal breast cancer cells, we performed transcriptional profiling of the MDA-MB-231 cells transduced with nontarget and MMP9 KD lentivirus, and found substantial alterations associated with MMP9 KD (Fig. 5A). We found that 1423 transcripts were differentially regulated (p<0.05, FC>2, annotated expression data in Supplemental Table 1). We subjected the list of differentially expressed genes to a NextBio meta-analysis [34], and found significant overlap with datasets comparing metastatic *vs.* nonmetastatic breast cancers (Fig. 5B; Supplemental Fig. 1; Supplemental Table 2), datasets comparing more *vs*. less advanced breast cancers (Fig. 5C; Supplemental Fig. 2; Supplemental Table 3), datasets comparing poorer *vs.* better prognosis breast cancers (Fig. 5D; Supplemental Fig. 3; Supplemental Table 4), datasets comparing basal subtype breast cancers *vs.* other subtypes (Fig. 5E; Supplemental Fig. 4; Supplemental Table 5), and datasets comparing ER- *vs.* ER+ breast cancers (Fig. 5F; Supplemental Fig. 5; Supplemental Table 6). These results suggest that the MMP9 that is expressed in these triple negative breast cancer cells activates a wide variety of pro-tumorigenic responses. Consistent with these results, Ingenuity Pathway Analysis (IPA) of the differentially expressed genes (Supplemental Table 7) identified a top-ranked interaction network that included a prominent nexus of genes associated with MMPs and stromal molecules (Fig. 5G; Supplemental Table 7). Using qRT/PCR, we validated transcriptional alterations in three of the most highly regulated genes in this network: the forkhead transcription factor FOXQ1 (Fig. 5H), the urokinase-type plasminogen activator (PLAU, Fig. 5I), and the BRCA1 interacting protein C-terminal helicase 1 (BRIP1, Fig. 5J).

### MMP9 is most highly expressed in Basal-like and ER-negative breast cancers

**Figure 6:**
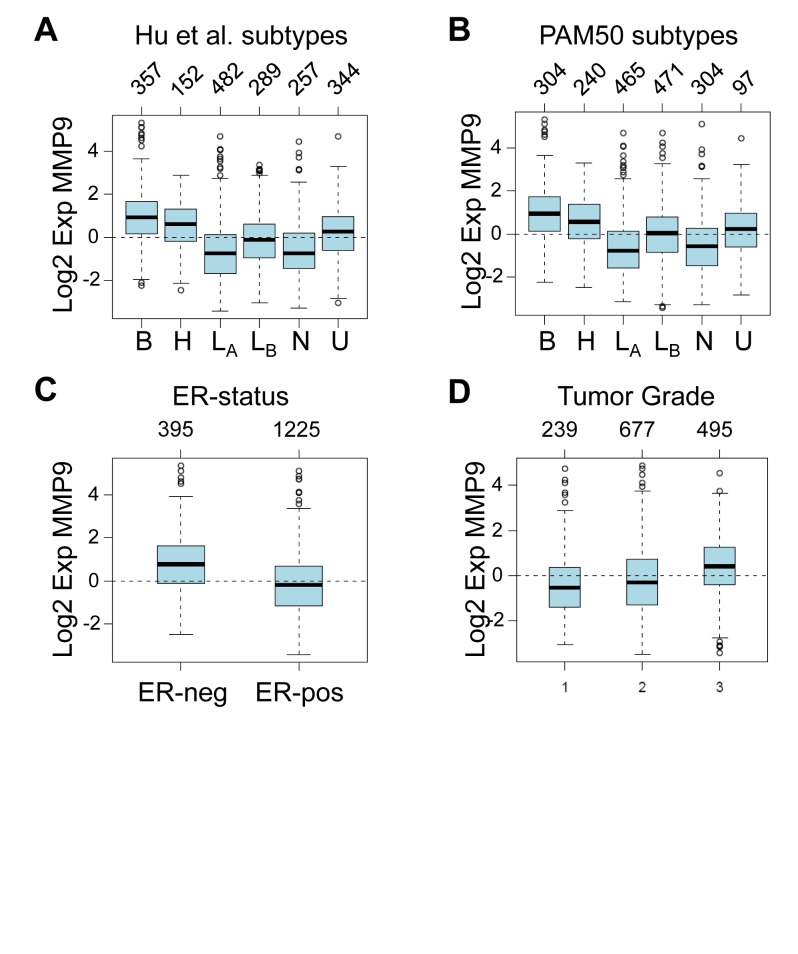
MMP9 is most highly expressed in basal-like, ER-negative, and high grade tumors (A) Box plot of MMP9 expression in 1881 human breast tumor samples stratified according to Hu *et al.* subtypes [36]. Number of tumors per group is indicated above the plot. Tumor classifications: B, basal; H, HER2 enriched; LA, luminal A; LB, luminal B; N, normal-like; U, unclassified. (B) Box plot of MMP9 expression in breast tumors stratified according to PAM50 subtypes [37]; tumor classifications are abbreviated as above. (C) Box plot of MMP9 expression in breast tumors stratified according to ER status. (D) Box plot of MMP9 expression in breast tumors stratified according to histological grade. All plots represent meta-analyses derived using GOBO: Gene Expression-Based Outcome for Breast Cancer Online [35], and included 1,881 patients from 11 distinct studies. For each plot, MMP9 expression differences between groups is highly significant (p<0.00001).

Given our results supporting a role for tumor cell-produced MMP9 in the invasion and metastasis of cell lines derived from human basal-like triple negative breast tumors, we hypothesized that MMP9 may be particularly highly expressed in these types of cancers. By interrogating a large meta-analysis of published breast cancer microarray datasets [35], we found that MMP9 expression was most highly elevated in basal-like breast cancers, as classified according to the intrinsic subtypes of Hu *et al.* [36] (Fig. 6A) or the PAM50 subtypes [37] (Fig. 6B), when compared to other molecular subtypes (p<0.00001). This analysis also revealed that estrogen receptor (ER) negative tumors express a significantly higher level of MMP9 than do ER positive tumors (p<0.00001) (Fig. 6C). In assessing a potential association between MMP9 and tumor grade, we found that MMP9 expression was associated with high grade tumors (p= <0.00001) (Fig. 6D). These results, in combination with our findings using cell culture and mouse models to functionally implicate tumor cell-produced MMP9 in invasion and metastasis, suggest that the aggressive phenotype and poor outcome generally associated with basal-like triple negative breast cancers [8, 10] are in part driven by MMP9. Taken together, our results suggest MMP9 as a potential target for antimetastatic therapies for this particular patient subset.

**Table 1P:** Overlapping genesets identified through meta-analysis

**Overlapping genesets**	**p-value**	**reference**
**Metastatic breast cancer**		
BC mets vs no mets	2.9E-13	[79]
BC N2 vs N0	2.1E-14	[80]
BC node pos vs node neg	1.1E-5	[81]
Strongly lung met vs parental	7.5E-21	[82]
Strongly lung met vs parental	3.3E-17	[83]
**Overlapping genesets**	**p-value**	**reference**
**Stage 3 vs Stage 1**		
SBR grade 3 vs 1	2.6E-29	[79]
SBR grade 3 vs 1	1.5E-26	[84]
SBR grade 3 vs 1	1.1E-23	[85]
Elston grade 3 vs 1	7.1E-22	[81]
Elston grade 3 vs 1	3.3E-21	[86]
Elston grade 3 vs 1	3.8E-21	[87]
Grade 3 vs 1	9.7E-24	[88]
Grade 3 vs 1	1.4E-24	[89]
Grade 3 vs 1	2.1E-23	[90]
Grade 3 vs 1	1.9E-20	[91]
Grade 3 vs 1	6.8E-14	[92]
Grade 3 vs 1	1.2E-7	[93]
**Overlapping genesets**	**p-value**	**reference**
**Poor prognosis**		
DFS 2 yr vs >4 yr	4.5E-22	[84]
DFS <31 mo vs >72 mo	1.6E-20	[92]
OS <5yr vs >10 yr	8.1E-20	[81]
Tumor >2.5cm vs <2.5cm	4.0E-9	[90]
Tumor >2.5 cm vs <2.0 cm	7.3E-12	[81]
Bad Van’t Veer vs good	2.7E-7	[83]
**Overlapping genesets**	**p-value**	**reference**
**Basal vs other subtypes**		
Basal vs Luminal A	1.7E-23	[89]
Basal vs normal-like subtype	5.8E-27	[85]
Basal vs normal-like subtype	1.9E-21	[87]
Basal vs normal-like subtype	6.1E-15	[93]
Basal BC vs non-basal BC	2.7E-13	[94]
Basal BC vs normal tissue	5.6E-20	[84]
**Overlapping genesets**	**p-value**	**reference**
**ER- vs ER+**		
ER neg vs ER pos	5.1E-19	[89]
ER neg vs ER pos	2.9E-17	[90]
ER neg vs ER pos	1.4E-12	[86]
ER neg vs ER pos	2.5E-8	[93]
ER/PR neg vs pos	2.2E-23	[94]
ER/PR neg vs pos	1.1E-12	[86]
Triple negative vs triple pos	2.5E-15	[94]
Triple negative vs triple pos	2.1E-7	[80]

## DISCUSSION

MMPs as a group are often considered to derive mainly from the stroma, although immunohistochemical and *in situ* hybridization studies show considerable diversity in staining patterns among the MMPs. In breast cancer, multiple large IHC studies have found MMP9 to be most consistently expressed by tumor cells, whereas MMP9 staining of stromal cells is rarer [23-26]. However, the functional implications for progression and metastasis of tumor cell-produced MMP9 have remained obscure, as previous experimental studies measuring metastasis as an endpoint have employed transgenic models best suited for probing the role of stromal cell-produced MMP9. In this study, we have identified MMP9 expression by tumor cells themselves as a critical factor mediating the invasion and metastasis of basal-like triple negative breast cancers. In our *in vivo* model, the impact of MMP9 knockdown in tumor cells on spontaneous metastasis was striking, resulting in a complete blockade of pulmonary metastasis. Consistent with prior studies implicating MMP9 in angiogenesis and vasculogenesis, we also found that tumor cell-produced MMP9 was a significant contributor to the vascularization of the orthotopic tumors, although the impact of MMP9 knockdown on primary tumor growth was surprisingly modest. It may be that the fewer blood vessels present in the MMP9 knockdown tumors were still functional and sufficient to support tumor growth.

The source of MMP9 found in human breast tumors has been seen to have significant bearing on its prognostic interpretation. Studies that evaluated the prognostic value of stromal MMP9 found significant associations with shorter recurrence-free survival [24, 25], while the prognostic interpretation of tumor cell MMP9 has remained more ambiguous. Some studies have found such expression to be a negative prognostic factor [23, 25, 27, 38], while others have reported associations between MMP9 expression and more favorable outcomes in certain patient subgroups [24, 26]. Our present findings suggest triple negative breast cancer specifically as a focus for future clinical studies examining the prognostic significance of tumor cell MMP9, as it may be that the pathways by which tumor MMP9 drives progression and metastasis are most activated or most necessary for this breast cancer subtype.

Why should the cell from which MMP9 is derived be important for its role in cancer progression or for its prognostic significance? One possibility is that tumor cell MMP9 may carry out specialized functions made possible by specific localization, through generation of high local concentrations at a key point of action. Furthermore, although a soluble secreted enzyme, MMP9 possesses protein-protein binding domains that can serve to tether it to the cancer cell surface, facilitating spatially discrete and directional activities [39]. MMP9 can be localized to the surface of murine mammary carcinoma cells via the hyaluronan receptor CD44 [40], as a component of tumor cell invadopodia structures [41], where it can facilitate invasion and angiogenesis by proteolytic activation of latent TGF-β [42]. CD44-bound MMP9 may also possess migration-promoting signaling activities that are distinct from its proteolytic activity [43]. In other types of tumor cells, binding of MMP9 to specific integrins can promote cell migration [44, 45]. Further studies will be needed to unravel the mechanisms by which tumor cell-produced MMP9 drives invasion and metastatic progression specifically in models of basal-like triple negative breast cancer.

As a first step toward elucidating the mechanisms by which basal-like triple negative cancer cell MMP9 is involved in invasion and metastasis, we carried out transcriptional profiling to compare MDA-MB-231 cells expressing endogenous levels of MMP9 *versus* cells in which MMP9 expression was silenced by RNA interference. MMP9 silencing resulted in broad and comprehensive transcriptional alterations, suggesting that the blockade of metastasis results from a complex transformation in cellular phenotype from a more malignant to a less malignant state. MMP9 has primarily been viewed as a direct effector of cellular motility, both through cleavage of ECM molecules and of cell-cell and cell-ECM attachments. Our results suggest that the autologous effects of MMP9 on the intrinsic breast cancer cell phenotype may be even more far-reaching, that MMP9 may act upstream of many genes as a master regulator of the malignant phenotype. We identified and validated several known metastasis mediators as regulated by MMP9 (Fig. 5), including Foxq1 [46] and PLAU [47], as well as the suspected breast cancer susceptibility gene BRIP1 [48]. The mechanisms by which MMP9 orchestrates this comprehensive phenotypic switch are likely to involve outside-in cell surface signal transduction, where the role of MMP9 has been increasingly appreciated in recent years [49].

Breast cancer prognosis varies widely among subtypes, and while outcomes have improved in recent years for estrogen receptor (ER)-expressing and HER2-overexpressing tumors, similar gains have not been achieved for patients with triple negative breast cancers lacking these molecular targets [9], most of which also belong to the poor prognosis basal-like intrinsic subtype [10, 50]. For patients with triple negative disease, the majority will have residual disease after initial treatment, a high risk of metastatic relapse within the first few years, and a median survival of only 1 year after relapse [8, 9]. Treatment options for these patients are limited following progression after standard chemotherapy regimens [8, 9], and new molecular targets and targeted therapies are urgently needed. Our data implicating tumor cell-produced MMP9 as a molecular driver of metastatic progression in models of basal-like triple negative breast cancer, in combination with the meta-analyses showing that MMP9 is most highly upregulated in these types of tumors, suggests MMP9 as a druggable target that may prove useful in these patients for whom established targeted therapies are of minimal benefit.

MMPs are not new as therapeutic targets, and past clinical trials of early MMP inhibitors proved disappointing [51, 52]. The broad-spectrum MMP inhibitors previously trialed in breast cancer produced serious dose-limiting musculoskeletal toxicity, failed to reach therapeutic plasma levels, and did not extend survival [53-55], likely due in part to the inhibitors' inability to distinguish among MMPs [56, 57]. This was a critical problem, because some MMPs serve a primarily protective function, and indiscriminate inhibition of all MMPs can lead to poorer outcomes [58-61]. In the decade that has passed since the abandonment of early generation MMP inhibitors as experimental cancer drugs, many novel and innovative approaches to more selective MMP9 inhibition have emerged. These include a new family of mechanism-based small molecule inhibitors of gelatinases [62, 63], as well as peptides and small molecules that inhibit subsets of MMP9 substrate or ligand interactions by blocking exosites on the collagen-binding domain [45, 64] or hemopexin domain [18, 45, 65, 66]. Candidates for development of therapeutic proteins include neutralizing monoclonal antibodies targeting the MMP9 catalytic domain [67, 68], and recombinant engineered versions of natural tissue inhibitors of metalloproteinases (TIMPs) [29, 69]. Recent comparative structural analyses of MMPs and TIMPs highlight new concepts underlying molecular specificity and offer promise for future development of yet more selective biologic inhibitors [70-74]. As future mechanistic studies reveal in greater detail the specific targets or interactions of tumor cell MMP9 that are essential for driving metastasis in basal-like triple negative cancers, it is anticipated that an optimal inhibitory approach can be selected and carried forward into clinical development, for the potential benefit of patients suffering from this challenging disease.

## MATERIALS AND METHODS

### Cell culture

MDA-MB-231-luc2 cells strain D3H1 (Caliper Life Science, Hopkinton, MA) were grown in EMEM media with 10% heat-inactivated fetal bovine serum (FBS) without antibiotics at 37 °C in 5% CO_2_. SUM159PT cells (generous gift from Dr. Stephen P. Ethier, Medical University of South Carolina) were grown in Ham's F-12 media with 5% FBS, 10 mM HEPES, 5 μg/mL insulin and 1 μg/mL hydrocortisone at 37 °C in 10% CO_2_. BT-549 cells were grown in RPMI 1640 media supplemented with 10% heat-inactivated FBS and 1% gentamicin at 37 °C in 5% CO_2_.

### Virus production and transduction

Lentiviral short hairpin RNA constructs NM_004994.1-2015s1c1 (HF19) and NM_004994.1-884s1c1 (HF22) targeting human MMP9 and NM_004530.1-496s1c1 targeting MMP2 were obtained from the MISSION TRC-Hs1.0 library (Sigma). A nontarget (NT) lentiviral vector containing a short hairpin that does not recognize any human genes was used as a negative control in all RNAi experiments. In experiments that employed a single MMP9 knockdown virus, NM_004994.1-2015s1c1 (HF19) was used.

Conditioned media containing infective lentivirus particles were produced using HEK 293FT cells following supplier protocols. For lentiviral transduction, MDA-MB-231-luc2, SUM159PT, or BT-549 cancer cells were seeded in 6 well plates, and then 0.6 mL regular medium, 0.4 ml of lentiviral medium and 0.6 μL of polybrene (10 mg/mL) were added. The medium was changed after 24 h and after another 24 h cultures were subjected to puromycin selection. Pooled transduced cells were maintained under puromycin selection for up to a week before use in experiments.

### RNA extraction, cDNA synthesis, and quantitative real-time PCR

RNA was isolated from cultured cells using TRIZol reagent (Invitrogen) according to manufacturer protocols. cDNA was synthesized according to kit specifications using the High Capacity cDNA Reverse Transcription Kit (Applied Biosystems, Foster City, CA). Quantitative real-time PCR was performed using TaqMan gene expression assays (Applied Biosystems) on an Applied Biosystems 7900HT Fast Real-Time PCR System according to manufacturer protocols. TaqMan assays employed included: GAPDH, Hs99999905_m1; MMP9, Hs00957555_m1; MMP2, Hs00234422_m1; FOXQ1, Hs00536425_s1; PLAU, Hs00170182_m1; and BRIP1, Hs00230743_m1. Data were analyzed using SDS RQ Manager Software (Applied Biosystems), using GAPDH as an endogenous control for normalization.

### Matrigel transwell invasion assays

Cellular invasion assays were similar to our previously published protocols [29, 75] with minor modifications. Briefly, BD Falcon 24-well cell culture inserts (8.0 μm) were coated with 50 μg Matrigel basement membrane matrix in 100 μL of serum free medium appropriate for the cell type (EMEM for MDA-MB-231-luc2 cells; Ham F-12 media for SUM159PT cells; RPMI 1640 for BT-549 cells) and placed at 37 °C for 4 h, and then residual medium was aspirated and replaced with cells (2.5×10^4^ MDA-MB-231-luc2 or BT-549 cells or 5×10^4^ SUM159PT cells per well) suspended in 300 μL of the appropriate serum free medium supplemented with 0.1% BSA. The lower invasion chambers contained 750 μL/well of NIH/3T3 cell-conditioned serum free medium (DMEM supplemented with 50 μg/mL ascorbic acid) as chemo-attractant. Assays were incubated 18 hours at 37°C in 5% CO_2_. Non-invading cells were removed from the insert by scrubbing with a cotton swab, and then cells on the lower surface of the filter were fixed with methanol, stained with crystal violet, and counted using Image-Pro 6.3 software (Media Cybernetics) as previously described [75].

### Mouse orthotopic tumor model and bioluminescence imaging

Animal Studies were conducted in accordance with recommended guidelines and approval of the Mayo Clinic Institutional Animal Care and Use Committee (IACUC) (protocol A12409). Prepared MDA-MB-231-luc2 cells (1×10^5^ cells per animal), in a total volume of 100 μl of 50% Matrigel/50% medium per animal, were kept on ice until surgical implantation. Six week old female Nod/Scid mice were anesthetized and a small ventral incision was made to visualize the left 4^th^ mammary fad pat. Cells were implanted at the blood vessel bifurcation near the lymph node. Tumor growth and metastasis were monitored each week over the time course by intraperitoneal injection with 150 mg/kg of D-luciferin and bioluminescence imaging (IVIS Spectrum 3D imaging system, Caliper Life Sciences) essentially as described previously [75]. At 11 weeks mice were injected intraperitoneally with luciferin and euthanized by CO_2_ asphyxiation, and then the tumors and lungs were excised, imaged *ex vivo* for bioluminescence signal, and formalin fixed and paraffin embedded for immunohistochemistry staining.

### Immunohistochemistry

Sectioned tumors were mounted and stained with hematoxylin and eosin, human cytokeratin antibody clones AE1/AE3 (Dako North America), MMP9 antibody #3852 (Cell Signaling), and CD31 antibody (PECAM-1 M-20; Santa Cruz #sc-1506). Slides were scanned and analyzed using Aperio ImageScope Software algorithms for staining intensity (MMP9) and number of positive cell groups (CD31).

### Transcriptional microarray and NextBio analysis

Isolated total RNA was assessed with Affymetrix U130A gene expression chips which were processed and normalized via GCRMA. Duplicate technical replicates were averaged and then analyzed using Genespring GX via t-tests using previously described methods [76, 77]. Thus, results presented are averages of two separate experiments performed in duplicate. Nextbio (www.nextbio.com) meta-analysis [34] was performed to identify similarities between our dataset and published datasets as previously described [78]. Ingenuity Pathway Analysis (IPA) was performed using the web interface (www.ingenuity.com) to build gene interactions associated with expression of MMP9.

### Meta-analysis of human breast cancer expression data

The online platform Gene expression-based Outcome for Breast cancer Online (GOBO), including data from 1,881 patients and 11 studies employing Affimetrix U133A microarrays, was used for meta-analysis of associations between MMP9 expression and other histopathological and molecular classifications (http://co.bmc.lu.se/gobo/) [35].

## SUPPLEMENTARY FIGURES AND TABLES















## References

[1] Siegel R, Naishadham D, Jemal A (2013). Cancer statistics, 2013. CA Cancer J Clin.

[2] Perou CM, Sorlie T, Eisen MB, van de Rijn M, Jeffrey SS, Rees CA, Pollack JR, Ross DT, Johnsen H, Akslen LA, Fluge O, Pergamenschikov A, Williams C, Zhu SX, Lonning PE, Borresen-Dale AL (2000). Molecular portraits of human breast tumours. Nature.

[3] van ‘t Veer LJ, Dai H, van de Vijver MJ, He YD, Hart AA, Mao M, Peterse HL, van der Kooy K, Marton MJ, Witteveen AT, Schreiber GJ, Kerkhoven RM, Roberts C, Linsley PS, Bernards R, Friend SH (2002). Gene expression profiling predicts clinical outcome of breast cancer. Nature.

[4] Wang Y, Klijn JG, Zhang Y, Sieuwerts AM, Look MP, Yang F, Talantov D, Timmermans M, Meijer-van Gelder ME, Yu J, Jatkoe T, Berns EM, Atkins D, Foekens JA (2005). Gene-expression profiles to predict distant metastasis of lymph-node-negative primary breast cancer. Lancet.

[5] Blows FM, Driver KE, Schmidt MK, Broeks A, van Leeuwen FE, Wesseling J, Cheang MC, Gelmon K, Nielsen TO, Blomqvist C, Heikkila P, Heikkinen T, Nevanlinna H, Akslen LA, Begin LR, Foulkes WD (2010). Subtyping of breast cancer by immunohistochemistry to investigate a relationship between subtype and short and long term survival: a collaborative analysis of data for 10,159 cases from 12 studies. PLoS Medicine.

[6] Sainsbury R (2013). The development of endocrine therapy for women with breast cancer. Cancer Treat Rev.

[7] Romond EH, Perez EA, Bryant J, Suman VJ, Geyer CE, Davidson NE, Tan-Chiu E, Martino S, Paik S, Kaufman PA, Swain SM, Pisansky TM, Fehrenbacher L, Kutteh LA, Vogel VG, Visscher DW (2005). Trastuzumab plus Adjuvant Chemotherapy for Operable HER2-Positive Breast Cancer. N Engl J Med.

[8] Andre F, Zielinski CC (2012). Optimal strategies for the treatment of metastatic triple-negative breast cancer with currently approved agents. Ann Oncol.

[9] Bayraktar S, Gluck S (2013). Molecularly targeted therapies for metastatic triple-negative breast cancer. Breast Cancer Res Treat.

[10] Bertucci F, Finetti P, Birnbaum D (2012). Basal breast cancer: a complex and deadly molecular subtype. Current Molecular Medicine.

[11] Dent R, Trudeau M, Pritchard KI, Hanna WM, Kahn HK, Sawka CA, Lickley LA, Rawlinson E, Sun P, Narod SA (2007). Triple-negative breast cancer: clinical features and patterns of recurrence. Clin Cancer Res.

[12] Stamenkovic I (2003). Extracellular matrix remodelling: the role of matrix metalloproteinases. J Pathol.

[13] Parks WC, Wilson CL, Lopez-Boado YS (2004). Matrix metalloproteinases as modulators of inflammation and innate immunity. Nat Rev Immunol.

[14] Kessenbrock K, Plaks V, Werb Z (2010). Matrix metalloproteinases: regulators of the tumor microenvironment. Cell.

[15] Gialeli C, Theocharis AD, Karamanos NK (2011). Roles of matrix metalloproteinases in cancer progression and their pharmacological targeting. FEBS Journal.

[16] Radisky ES, Radisky DC (2010). Matrix metalloproteinase-induced epithelial-mesenchymal transition in breast cancer. J Mammary Gland Biol Neoplasia.

[17] Radisky ES, Radisky DC (2007). Stromal induction of breast cancer: inflammation and invasion. Rev Endocr Metab Disord.

[18] Dufour A, Sampson NS, Li J, Kuscu C, Rizzo RC, Deleon JL, Zhi J, Jaber N, Liu E, Zucker S, Cao J (2011). Small-molecule anticancer compounds selectively target the hemopexin domain of matrix metalloproteinase-9. Cancer Res.

[19] McGowan PM, Duffy MJ (2008). Matrix metalloproteinase expression and outcome in patients with breast cancer: analysis of a published database. Ann Oncol.

[20] Acuff HB, Sinnamon M, Fingleton B, Boone B, Levy SE, Chen X, Pozzi A, Carbone DP, Schwartz DR, Moin K, Sloane BF, Matrisian LM (2006). Analysis of host- and tumor-derived proteinases using a custom dual species microarray reveals a protective role for stromal matrix metalloproteinase-12 in non-small cell lung cancer. Cancer Res.

[21] Itoh T, Tanioka M, Matsuda H, Nishimoto H, Yoshioka T, Suzuki R, Uehira M (1999). Experimental metastasis is suppressed in MMP-9-deficient mice. Clin Exp Metastasis.

[22] Martin MD, Carter KJ, Jean-Philippe SR, Chang M, Mobashery S, Thiolloy S, Lynch CC, Matrisian LM, Fingleton B (2008). Effect of ablation or inhibition of stromal matrix metalloproteinase-9 on lung metastasis in a breast cancer model is dependent on genetic background. Cancer Res.

[23] Li HC, Cao DC, Liu Y, Hou YF, Wu J, Lu JS, Di GH, Liu G, Li FM, Ou ZL, Jie C, Shen ZZ, Shao ZM (2004). Prognostic value of matrix metalloproteinases (MMP-2 and MMP-9) in patients with lymph node-negative breast carcinoma. Breast Cancer Res Treat.

[24] Pellikainen JM, Ropponen KM, Kataja VV, Kellokoski JK, Eskelinen MJ, Kosma VM (2004). Expression of matrix metalloproteinase (MMP)-2 and MMP-9 in breast cancer with a special reference to activator protein-2, HER2, and prognosis. Clin Cancer Res.

[25] Vizoso FJ, Gonzalez LO, Corte MD, Rodriguez JC, Vazquez J, Lamelas ML, Junquera S, Merino AM, Garcia-Muniz JL (2007). Study of matrix metalloproteinases and their inhibitors in breast cancer. Br J Cancer.

[26] Scorilas A, Karameris A, Arnogiannaki N, Ardavanis A, Bassilopoulos P, Trangas T, Talieri M (2001). Overexpression of matrix-metalloproteinase-9 in human breast cancer: a potential favourable indicator in node-negative patients. Br J Cancer.

[27] Hao L, Zhang C, Qiu Y, Wang L, Luo Y, Jin M, Zhang Y, Guo TB, Matsushima K (2007). Recombination of CXCR4, VEGF, and MMP-9 predicting lymph node metastasis in human breast cancer. Cancer Lett.

[28] Wu ZS, Wu Q, Yang JH, Wang HQ, Ding XD, Yang F, Xu XC (2008). Prognostic significance of MMP-9 and TIMP-1 serum and tissue expression in breast cancer. Int J Cancer.

[29] Batra J, Robinson J, Mehner C, Hockla A, Miller E, Radisky DC, Radisky ES (2012). PEGylation extends circulation half-life while preserving in vitro and in vivo activity of tissue inhibitor of metalloproteinases-1 (TIMP-1). PloS One.

[30] Neve RM, Chin K, Fridlyand J, Yeh J, Baehner FL, Fevr T, Clark L, Bayani N, Coppe JP, Tong F, Speed T, Spellman PT, DeVries S, Lapuk A, Wang NJ, Kuo WL (2006). A collection of breast cancer cell lines for the study of functionally distinct cancer subtypes. Cancer Cell.

[31] Kao J, Salari K, Bocanegra M, Choi YL, Girard L, Gandhi J, Kwei KA, Hernandez-Boussard T, Wang P, Gazdar AF, Minna JD, Pollack JR (2009). Molecular profiling of breast cancer cell lines defines relevant tumor models and provides a resource for cancer gene discovery. PloS One.

[32] Bergers G, Brekken R, McMahon G, Vu TH, Itoh T, Tamaki K, Tanzawa K, Thorpe P, Itohara S, Werb Z, Hanahan D (2000). Matrix metalloproteinase-9 triggers the angiogenic switch during carcinogenesis. Nat Cell Biol.

[33] Ahn GO, Brown JM (2008). Matrix metalloproteinase-9 is required for tumor vasculogenesis but not for angiogenesis: role of bone marrow-derived myelomonocytic cells. Cancer Cell.

[34] Kupershmidt I, Su QJ, Grewal A, Sundaresh S, Halperin I, Flynn J, Shekar M, Wang H, Park J, Cui W, Wall GD, Wisotzkey R, Alag S, Akhtari S, Ronaghi M (2010). Ontology-based meta-analysis of global collections of high-throughput public data. PloS One.

[35] Ringnér M, Fredlund E, Häkkinen J, Borg Å, Staaf J (2011). GOBO: Gene Expression-Based Outcome for Breast Cancer Online. PLoS One.

[36] Hu Z, Fan C, Oh DS, Marron JS, He X, Qaqish BF, Livasy C, Carey LA, Reynolds E, Dressler L, Nobel A, Parker J, Ewend MG, Sawyer LR, Wu J, Liu Y (2006). The molecular portraits of breast tumors are conserved across microarray platforms. BMC Genomics.

[37] Parker JS, Mullins M, Cheang MC, Leung S, Voduc D, Vickery T, Davies S, Fauron C, He X, Hu Z, Quackenbush JF, Stijleman IJ, Palazzo J, Marron JS, Nobel AB, Mardis E (2009). Supervised risk predictor of breast cancer based on intrinsic subtypes. J Clin Oncol.

[38] Wu ZS, Wu Q, Yang JH, Wang HQ, Ding XD, Yang F, Xu XC (2008). Prognostic significance of MMP-9 and TIMP-1 serum and tissue expression in breast cancer. International Journal of Cancer.

[39] Fridman R, Toth M, Chvyrkova I, Meroueh SO, Mobashery S (2003). Cell surface association of matrix metalloproteinase-9 (gelatinase B). Cancer Metastasis Rev.

[40] Yu Q, Stamenkovic I (1999). Localization of matrix metalloproteinase 9 to the cell surface provides a mechanism for CD44-mediated tumor invasion. Genes Dev.

[41] Bourguignon LY, Gunja-Smith Z, Iida N, Zhu HB, Young LJ, Muller WJ, Cardiff RD (1998). CD44v(3,8-10) is involved in cytoskeleton-mediated tumor cell migration and matrix metalloproteinase (MMP-9) association in metastatic breast cancer cells. J Cell Physiol.

[42] Yu Q, Stamenkovic I (2000). Cell surface-localized matrix metalloproteinase-9 proteolytically activates TGF-beta and promotes tumor invasion and angiogenesis. Genes Dev.

[43] Dufour A, Sampson NS, Zucker S, Cao J (2008). Role of the hemopexin domain of matrix metalloproteinases in cell migration. J Cell Physiol.

[44] Stefanidakis M, Bjorklund M, Ihanus E, Gahmberg CG, Koivunen E (2003). Identification of a negatively charged peptide motif within the catalytic domain of progelatinases that mediates binding to leukocyte beta 2 integrins. J Biol Chem.

[45] Bjorklund M, Heikkila P, Koivunen E (2004). Peptide inhibition of catalytic and noncatalytic activities of matrix metalloproteinase-9 blocks tumor cell migration and invasion. J Biol Chem.

[46] Zhang H, Meng F, Liu G, Zhang B, Zhu J, Wu F, Ethier SP, Miller F, Wu G (2011). Forkhead transcription factor foxq1 promotes epithelial-mesenchymal transition and breast cancer metastasis. Cancer Res.

[47] Weigelt B, Peterse JL, van ‘t Veer LJ (2005). Breast cancer metastasis: markers and models. Nat Rev Cancer.

[48] Easton DF, Pooley KA, Dunning AM, Pharoah PD, Thompson D, Ballinger DG, Struewing JP, Morrison J, Field H, Luben R, Wareham N, Ahmed S, Healey CS, Bowman R, Meyer KB, Haiman CA (2007). Genome-wide association study identifies novel breast cancer susceptibility loci. Nature.

[49] Bauvois B (2012). New facets of matrix metalloproteinases MMP-2 and MMP-9 as cell surface transducers: outside-in signaling and relationship to tumor progression. Biochim Biophys Acta.

[50] Penault-Llorca F, Viale G (2012). Pathological and molecular diagnosis of triple-negative breast cancer: a clinical perspective. Ann Oncol.

[51] Coussens LM, Fingleton B, Matrisian LM (2002). Matrix metalloproteinase inhibitors and cancer: trials and tribulations. Science.

[52] Fingleton B (2008). MMPs as therapeutic targets-Still a viable option?. Semin Cell Dev Biol.

[53] Sparano JA, Bernardo P, Stephenson P, Gradishar WJ, Ingle JN, Zucker S, Davidson NE (2004). Randomized phase III trial of marimastat versus placebo in patients with metastatic breast cancer who have responding or stable disease after first-line chemotherapy: Eastern Cooperative Oncology Group trial E2196. J Clin Oncol.

[54] Miller KD, Gradishar W, Schuchter L, Sparano JA, Cobleigh M, Robert N, Rasmussen H, Sledge GW (2002). A randomized phase II pilot trial of adjuvant marimastat in patients with early-stage breast cancer. Ann Oncol.

[55] Miller KD, Saphner TJ, Waterhouse DM, Chen TT, Rush-Taylor A, Sparano JA, Wolff AC, Cobleigh MA, Galbraith S, Sledge GW (2004). A randomized phase II feasibility trial of BMS-275291 in patients with early stage breast cancer. Clin Cancer Res.

[56] Overall CM, Kleifeld O (2006). Towards third generation matrix metalloproteinase inhibitors for cancer therapy. Br J Cancer.

[57] Fisher JF, Mobashery S (2006). Recent advances in MMP inhibitor design. Cancer Metastasis Rev.

[58] Fingleton B (2006). Matrix metalloproteinases: roles in cancer and metastasis. Front Biosci.

[59] Martin MD, Matrisian LM (2007). The other side of MMPs: protective roles in tumor progression. Cancer Metastasis Rev.

[60] Overall CM, Kleifeld O (2006). Tumour microenvironment - opinion: validating matrix metalloproteinases as drug targets and anti-targets for cancer therapy. Nat Rev Cancer.

[61] Decock J, Thirkettle S, Wagstaff L, Edwards DR (2011). Matrix metalloproteinases: protective roles in cancer. J Cell Mol Med.

[62] Kruger A, Arlt MJ, Gerg M, Kopitz C, Bernardo MM, Chang M, Mobashery S, Fridman R (2005). Antimetastatic activity of a novel mechanism-based gelatinase inhibitor. Cancer Res.

[63] Ikejiri M, Bernardo MM, Bonfil RD, Toth M, Chang M, Fridman R, Mobashery S (2005). Potent mechanism-based inhibitors for matrix metalloproteinases. J Biol Chem.

[64] Lauer-Fields JL, Whitehead JK, Li S, Hammer RP, Brew K, Fields GB (2008). Selective modulation of matrix metalloproteinase 9 (MMP-9) functions via exosite inhibition. J Biol Chem.

[65] Dufour A, Zucker S, Sampson NS, Kuscu C, Cao J (2010). Role of matrix metalloproteinase-9 dimers in cell migration: design of inhibitory peptides. J Biol Chem.

[66] Ugarte-Berzal E, Bailon E, Amigo-Jimenez I, Vituri CL, del Cerro MH, Terol MJ, Albar JP, Rivas G, Garcia-Marco JA, Garcia-Pardo A (2012). A 17-residue sequence from the matrix metalloproteinase-9 (MMP-9) hemopexin domain binds alpha4beta1 integrin and inhibits MMP-9-induced functions in chronic lymphocytic leukemia B cells. J Biol Chem.

[67] Martens E, Leyssen A, Van Aelst I, Fiten P, Piccard H, Hu J, Descamps FJ, Van den Steen PE, Proost P, Van Damme J, Liuzzi GM, Riccio P, Polverini E, Opdenakker G (2007). A monoclonal antibody inhibits gelatinase B/MMP-9 by selective binding to part of the catalytic domain and not to the fibronectin or zinc binding domains. Biochim Biophys Acta.

[68] Sela-Passwell N, Kikkeri R, Dym O, Rozenberg H, Margalit R, Arad-Yellin R, Eisenstein M, Brenner O, Shoham T, Danon T, Shanzer A, Sagi I (2012). Antibodies targeting the catalytic zinc complex of activated matrix metalloproteinases show therapeutic potential. Nat Med.

[69] Hamze AB, Wei S, Bahudhanapati H, Kota S, Acharya KR, Brew K (2007). Constraining specificity in the N-domain of tissue inhibitor of metalloproteinases-1; gelatinase-selective inhibitors. Protein Sci.

[70] Sela-Passwell N, Rosenblum G, Shoham T, Sagi I (2010). Structural and functional bases for allosteric control of MMP activities: can it pave the path for selective inhibition?. Biochim Biophys Acta.

[71] Batra J, Robinson J, Soares AS, Fields AP, Radisky DC, Radisky ES (2012). Matrix metalloproteinase-10 (MMP-10) interaction with tissue inhibitors of metalloproteinases TIMP-1 and TIMP-2: binding studies and crystal structure. J Biol Chem.

[72] Batra J, Soares AS, Mehner C, Radisky ES (2013). Matrix Metalloproteinase-10/TIMP-2 Structure and Analyses Define Conserved Core Interactions and Diverse Exosite Interactions in MMP/TIMP Complexes. PLoS One.

[73] Udi Y, Fragai M, Grossman M, Mitternacht S, Arad-Yellin R, Calderone V, Melikian M, Toccafondi M, Berezovsky IN, Luchinat C, Sagi I (2013). Unraveling hidden regulatory sites in structurally homologous metalloproteases. J Mol Biol.

[74] Batra J, Radisky ES, Scott RA (2013). Tissue inhibitors of metalloproteinases (TIMPs): inhibition of Zn-dependent metallopeptidases. Encyclopedia of Inorganic and Bioinorganic Chemistry: John Wiley & Sons.

[75] Hockla A, Miller E, Salameh MA, Copland JA, Radisky DC, Radisky ES (2012). PRSS3/mesotrypsin is a therapeutic target for metastatic prostate cancer. Mol Cancer Res.

[76] Chen CS, Nelson CM, Khauv D, Bennett S, Radisky ES, Hirai Y, Bissell MJ, Radisky DC (2009). Homology with vesicle fusion mediator syntaxin-1a predicts determinants of epimorphin/syntaxin-2 function in mammary epithelial morphogenesis. J Biol Chem.

[77] Cichon MA, Gainullin VG, Zhang Y, Radisky DC (2012). Growth of lung cancer cells in three-dimensional microenvironments reveals key features of tumor malignancy. Integrative Biology.

[78] Stallings-Mann ML, Waldmann J, Zhang Y, Miller E, Gauthier ML, Visscher DW, Downey GP, Radisky ES, Fields AP, Radisky DC (2012). Matrix metalloproteinase induction of Rac1b, a key effector of lung cancer progression. Science Transl Med.

[79] Guedj M, Marisa L, de Reynies A, Orsetti B, Schiappa R, Bibeau F, MacGrogan G, Lerebours F, Finetti P, Longy M, Bertheau P, Bertrand F, Bonnet F, Martin AL, Feugeas JP, Bieche I (2012). A refined molecular taxonomy of breast cancer. Oncogene.

[80] Tabchy A, Valero V, Vidaurre T, Lluch A, Gomez H, Martin M, Qi Y, Barajas-Figueroa LJ, Souchon E, Coutant C, Doimi FD, Ibrahim NK, Gong Y, Hortobagyi GN, Hess KR, Symmans WF (2010). Evaluation of a 30-gene paclitaxel, fluorouracil, doxorubicin, and cyclophosphamide chemotherapy response predictor in a multicenter randomized trial in breast cancer. Clin Cancer Res.

[81] Miller LD, Smeds J, George J, Vega VB, Vergara L, Ploner A, Pawitan Y, Hall P, Klaar S, Liu ET, Bergh J (2005). An expression signature for p53 status in human breast cancer predicts mutation status, transcriptional effects, and patient survival. Proc Natl Acad Sci U S A.

[82] Lu X, Kang Y (2009). Efficient acquisition of dual metastasis organotropism to bone and lung through stable spontaneous fusion between MDA-MB-231 variants. Proc Natl Acad Sci U S A.

[83] Minn AJ, Gupta GP, Siegel PM, Bos PD, Shu W, Giri DD, Viale A, Olshen AB, Gerald WL, Massague J (2005). Genes that mediate breast cancer metastasis to lung. Nature.

[84] Sabatier R, Finetti P, Adelaide J, Guille A, Borg JP, Chaffanet M, Lane L, Birnbaum D, Bertucci F (2011). Down-regulation of ECRG4, a candidate tumor suppressor gene, in human breast cancer. PLoS One.

[85] Sircoulomb F, Bekhouche I, Finetti P, Adelaide J, Ben Hamida A, Bonansea J, Raynaud S, Innocenti C, Charafe-Jauffret E, Tarpin C, Ben Ayed F, Viens P, Jacquemier J, Bertucci F, Birnbaum D, Chaffanet M (2010). Genome profiling of ERBB2-amplified breast cancers. BMC Cancer.

[86] Ivshina AV, George J, Senko O, Mow B, Putti TC, Smeds J, Lindahl T, Pawitan Y, Hall P, Nordgren H, Wong JE, Liu ET, Bergh J, Kuznetsov VA, Miller LD (2006). Genetic reclassification of histologic grade delineates new clinical subtypes of breast cancer. Cancer Res.

[87] Pawitan Y, Bjohle J, Amler L, Borg AL, Egyhazi S, Hall P, Han X, Holmberg L, Huang F, Klaar S, Liu ET, Miller L, Nordgren H, Ploner A, Sandelin K, Shaw PM (2005). Gene expression profiling spares early breast cancer patients from adjuvant therapy: derived and validated in two population-based cohorts. Breast Cancer Res.

[88] Silver DP, Richardson AL, Eklund AC, Wang ZC, Szallasi Z, Li Q, Juul N, Leong CO, Calogrias D, Buraimoh A, Fatima A, Gelman RS, Ryan PD, Tung NM, De Nicolo A, Ganesan S (2010). Efficacy of neoadjuvant Cisplatin in triple-negative breast cancer. J Clin Oncol.

[89] Dedeurwaerder S, Desmedt C, Calonne E, Singhal SK, Haibe-Kains B, Defrance M, Michiels S, Volkmar M, Deplus R, Luciani J, Lallemand F, Larsimont D, Toussaint J, Haussy S, Rothe F, Rouas G (2011). DNA methylation profiling reveals a predominant immune component in breast cancers. EMBO Mol Med.

[90] Lu X, Wang ZC, Iglehart JD, Zhang X, Richardson AL (2008). Predicting features of breast cancer with gene expression patterns. Breast Cancer Res Treat.

[91] The International Genomics Consortium (IGC) The expO project (Expression Project for Oncology).

[92] Schmidt M, Bohm D, von Torne C, Steiner E, Puhl A, Pilch H, Lehr HA, Hengstler JG, Kolbl H, Gehrmann M (2008). The humoral immune system has a key prognostic impact in node-negative breast cancer. Cancer Res.

[93] Gluck S, Ross JS, Royce M, McKenna EF, Perou CM, Avisar E, Wu L (2012). TP53 genomics predict higher clinical and pathologic tumor response in operable early-stage breast cancer treated with docetaxel-capecitabine +/- trastuzumab. Breast Cancer Res Treat.

[94] Richardson AL, Wang ZC, De Nicolo A, Lu X, Brown M, Miron A, Liao X, Iglehart JD, Livingston DM, Ganesan S (2006). X chromosomal abnormalities in basal-like human breast cancer. Cancer Cell.

