# Supplementary material for: Tumor cell-produced matrix metalloproteinase 9 (MMP-9) drives malignant progression and metastasis of basal-like triple negative breast cancer
Source: Oncotarget. 2014 May 1;5(9):2736–49. doi: 10.18632/oncotarget.1932 (PMC4058041; doi:10.18632/oncotarget.1932)
Supplement: Supplementary file 1 [file oncotarget-05-2736-s001.pdf]

Tumor cell-produced matrix metalloproteinase 9 (MMP-9) drives malignant progression and metastasis of basal-like triple negative breast cancer

Supplemental Material

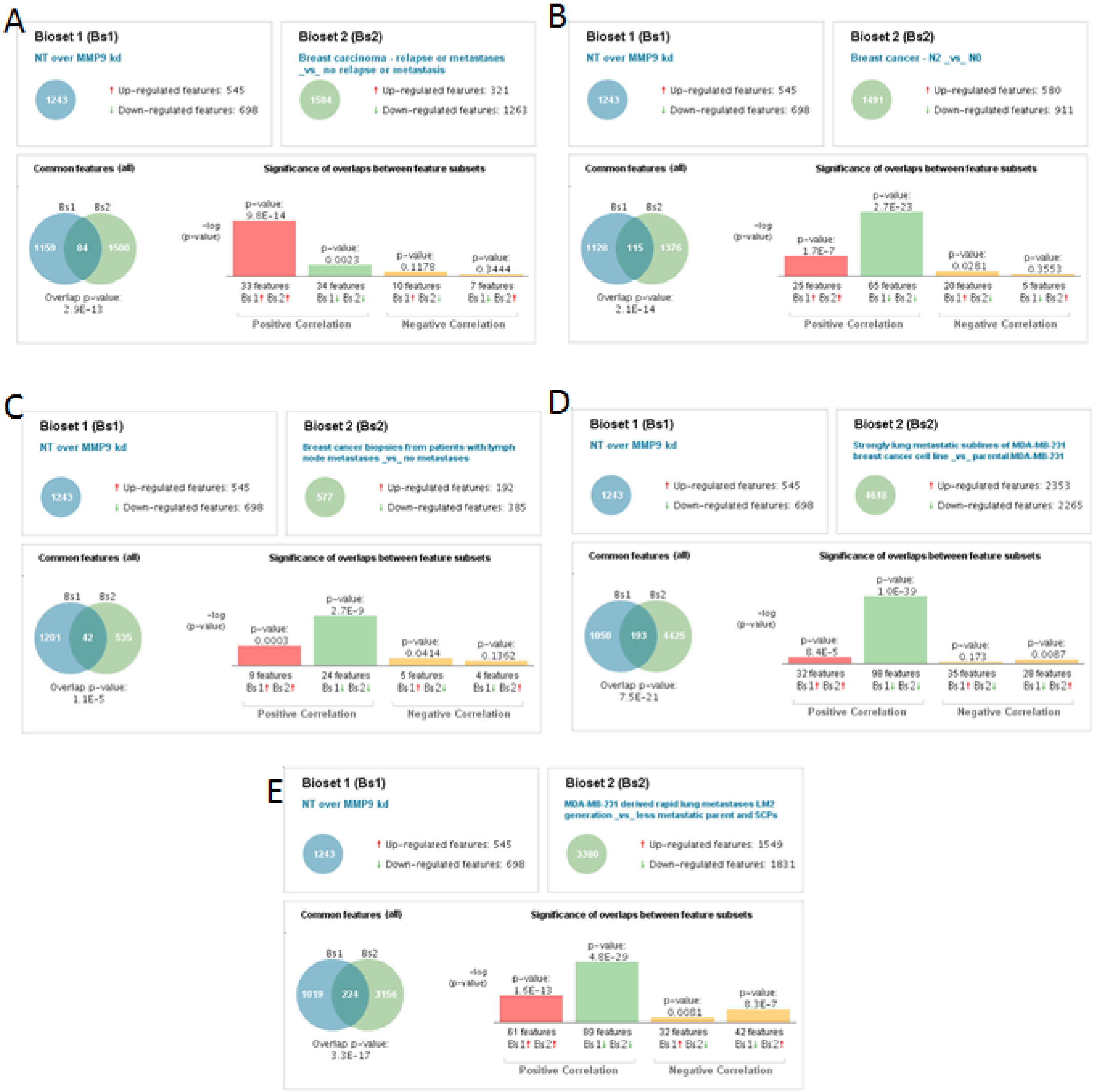

Supplemental Figure 1. Overlap with metastasis datasets. Overlap of significantly differentially regulated genes between MDAMB231 NT and MDAMB231 MMP9 KD and datasets comparing (A) BC mets vs no mets [1], (B) BC N2 vs N0 [2], (C) BC node pos vs node neg [3], (D) Strongly lung met vs parental [4], (E) Strongly lung met vs parental [5].

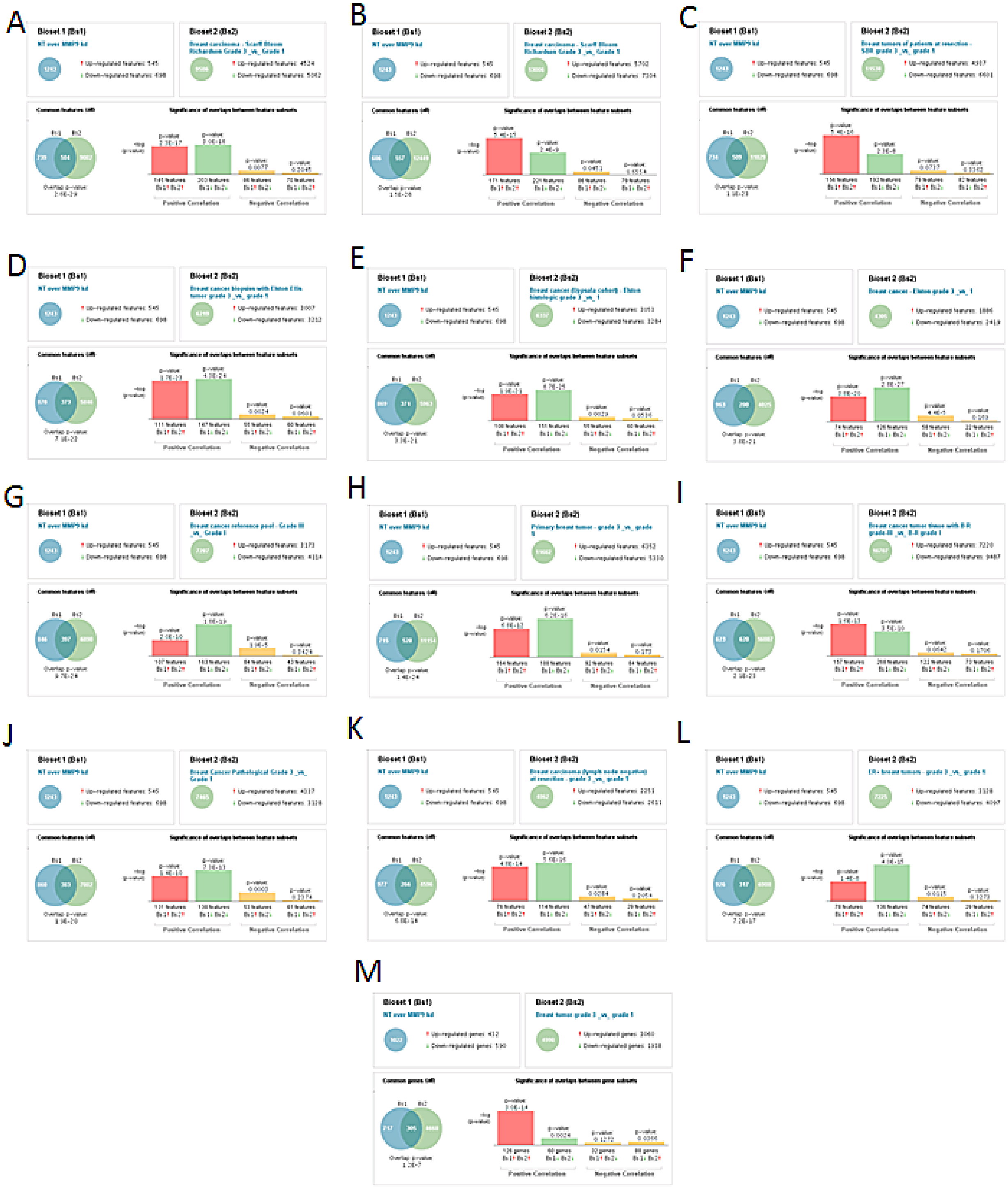

Supplemental Figure 2. Overlap with breast cancer grade 3 vs 1 datasets. Overlap of significantly differentially regulated genes between MDAMB231 NT and MDAMB231 MMP9 KD and datasets comparing (A) SBR grade 3 vs 1 [1], (B) SBR grade 3 vs 1 [6], (C) SBR grade 3 vs 1 [7], (D) Elston grade 3 vs 1 [3], (E) Elston grade 3 vs 1 [8], (F) Elston grade 3 vs 1 [9], (F)

Grade 3 vs 1 [10], (G) Grade 3 vs 1 [11], (G) Grade 3 vs 1 [12], (H) Grade 3 vs 1 [13], (I) Grade 3 vs 1 [14], (J) Grade 3 vs 1 [15].

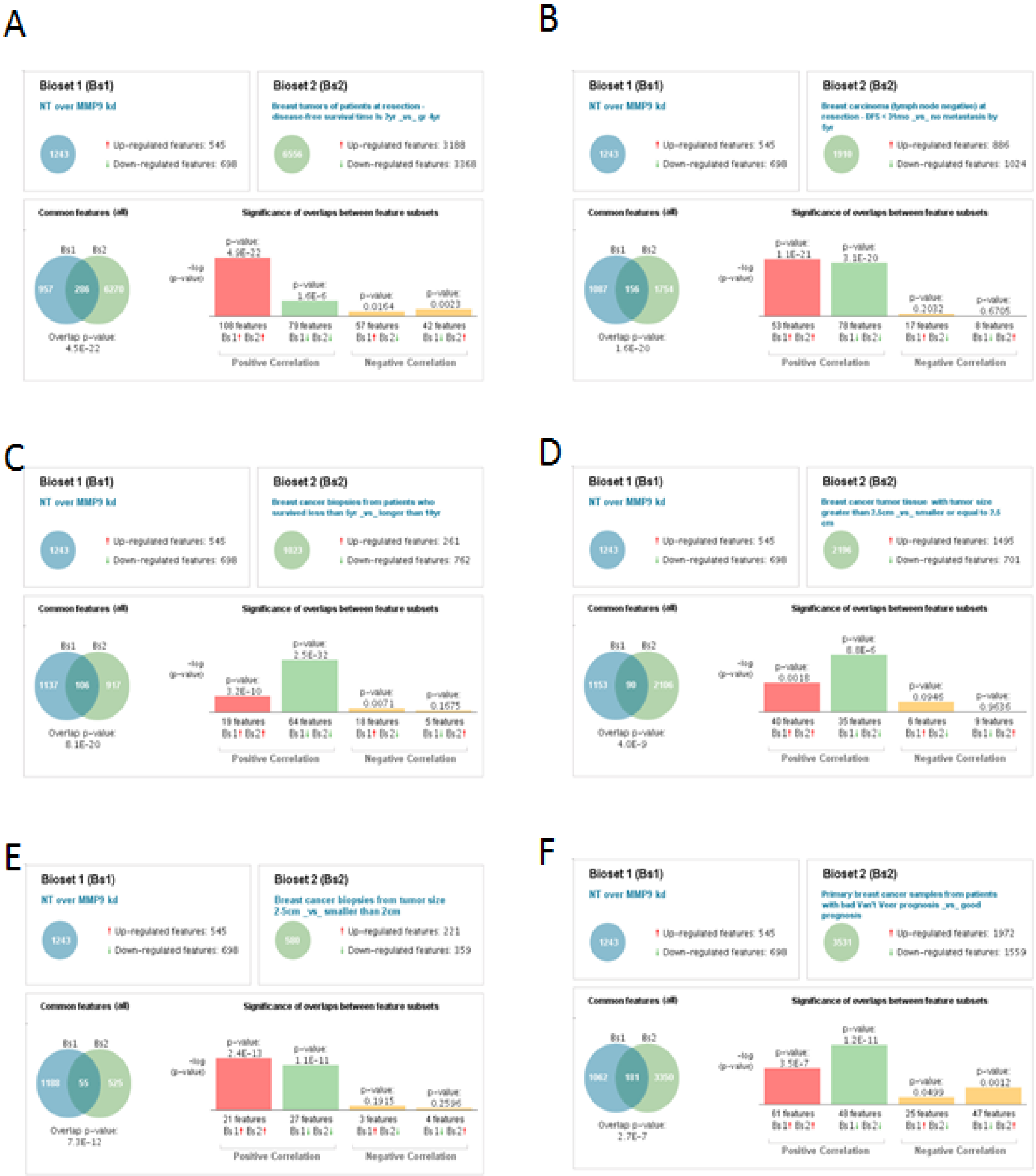

Supplemental Figure 3. Overlap with poor prognosis datasets. Overlap of significantly differentially regulated genes between MDAMB231 NT and MDAMB231 MMP9 KD and datasets comparing (A) disease-free survival 2 yr vs >4 yr [6], (B) disease-free survival <31 mo vs >72 mo [14], (C) overall survival <5yr vs >10 yr [3], (D) Tumor >2.5cm vs <2.5cm [12], (E) Tumor >2.5 cm vs <2.0 cm [3], (F) Bad Van't Veer vs good 2.7E-7 [5].

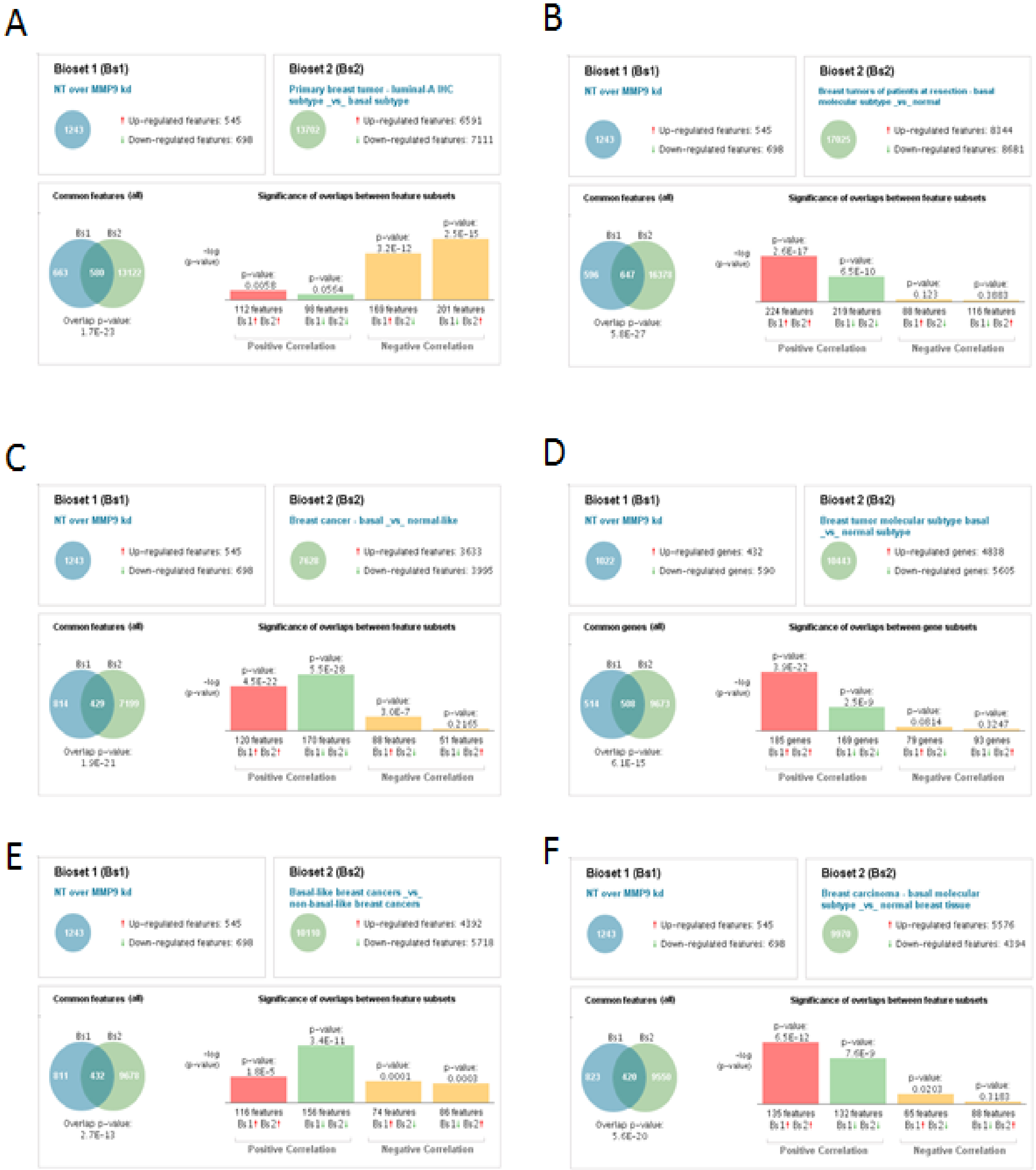

Supplemental Figure 4. Overlap with basal subtype vs other subtype datasets. Overlap of significantly differentially regulated genes between MDAMB231 NT and MDAMB231 MMP9 KD and datasets comparing (A) Basal vs Luminal A [11], (B) Basal vs normal-like subtype [7], (C) Basal vs normal-like subtype [9], (D) Basal vs normal-like subtype [15], (E) Basal BC vs non-basal BC [16], (F) Basal BC vs normal tissue [6].

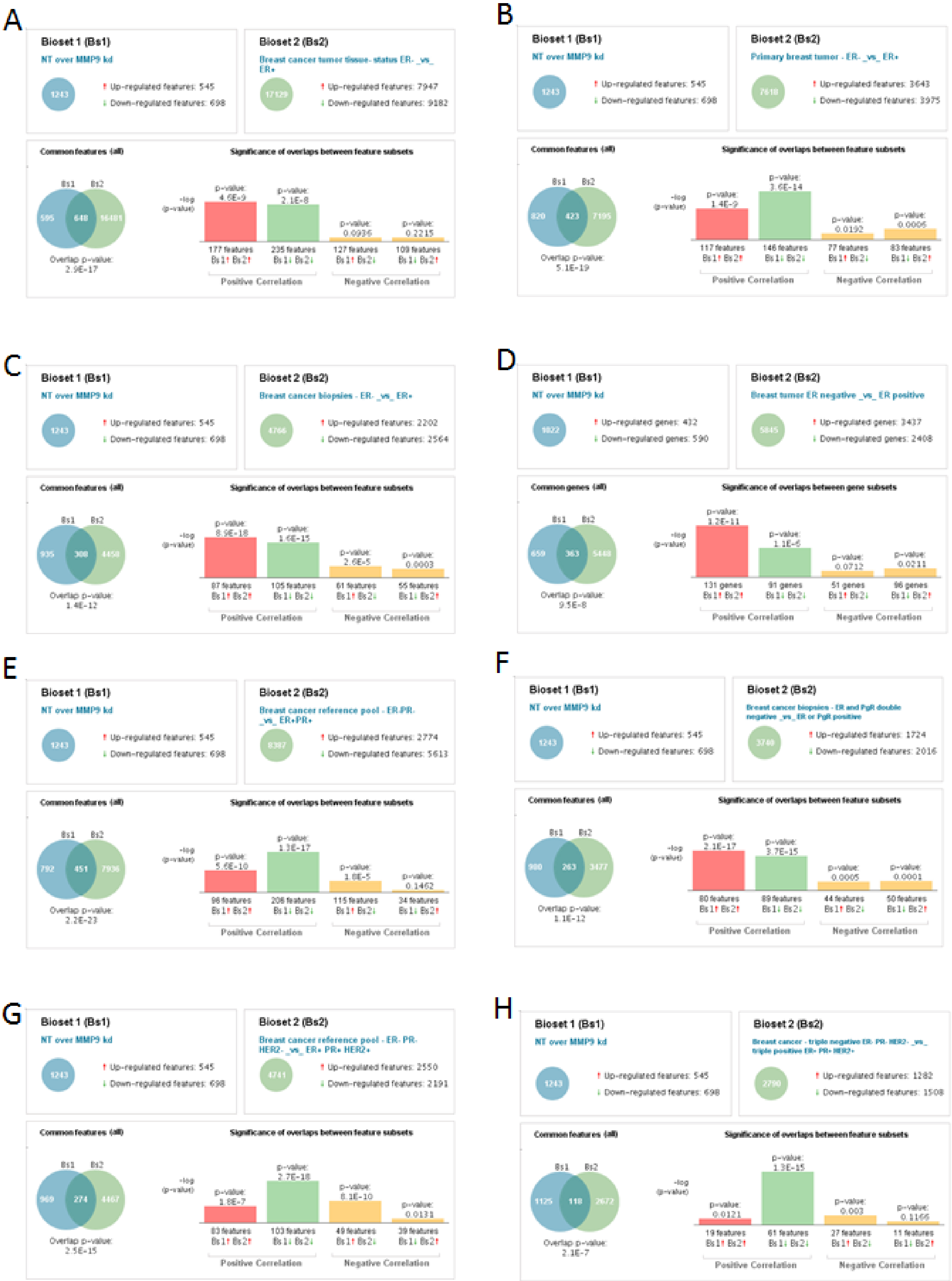
A
B
C
D
E
F
G
H
Bioset 1 (Bs1)
NT over MMP9 kd
Bioset 2 (Bs2)
Breast cancer tumor tissue- status ER- _vs_ ER+
Primary breast tumor - ER- _vs_ ER+
Breast cancer biopsies - ER- _vs_ ER+
Breast tumor ER negative _vs_ ER positive
Breast cancer reference pool - ER-PR- _vs_ ER+PR+
Breast cancer biopsies - ER and PgR double negative _vs_ ER or PgR positive
Breast cancer reference pool - ER- PR- HER2- _vs_ ER+ PR+ HER2+
Breast cancer - triple negative ER- PR- HER2- _vs_ triple positive ER+ PR+ HER2+
Up-regulated features
Down-regulated features
Common features (all)
Significance of overlaps between feature subsets
Overlap p-value
Positive Correlation
Negative Correlation

Supplemental Figure 5. Overlap with ER+ vs ER- datasets. Overlap of significantly differentially regulated genes between MDAMB231 NT and MDAMB231 MMP9 KD and datasets comparing (A) ER negative vs ER positive [11], (B) ER negative vs ER positive [12], (C) ER negative vs ER positive [8], (D) ER negative vs ER pos[15], (E) ER/PR negative vs positive [16], (F) ER/PR negative vs positive [8], (G) Triple negative vs triple positive [16], (H) Triple negative vs triple positive [2]

References

1. Guedj M, Marisa L, de Reynies A, Orsetti B, Schiappa R, Bibeau F, MacGrogan G, Lerebours F, Finetti P, Longy M *et al*: **A refined molecular taxonomy of breast cancer**. *Oncogene* 2012, **31**(9):1196-1206.
2. Tabchy A, Valero V, Vidaurre T, Lluch A, Gomez H, Martin M, Qi Y, Barajas-Figueroa LJ, Souchon E, Coutant C *et al*: **Evaluation of a 30-gene paclitaxel, fluorouracil, doxorubicin, and cyclophosphamide chemotherapy response predictor in a multicenter randomized trial in breast cancer**. *Clin Cancer Res* 2010, **16**(21):5351-5361.
3. Miller LD, Smeds J, George J, Vega VB, Vergara L, Ploner A, Pawitan Y, Hall P, Klaar S, Liu ET *et al*: **An expression signature for p53 status in human breast cancer predicts mutation status, transcriptional effects, and patient survival**. *Proc Natl Acad Sci U S A* 2005, **102**(38):13550-13555.
4. Lu X, Kang Y: **Efficient acquisition of dual metastasis organotropism to bone and lung through stable spontaneous fusion between MDA-MB-231 variants**. *Proc Natl Acad Sci U S A* 2009, **106**(23):9385-9390.
5. Minn AJ, Gupta GP, Siegel PM, Bos PD, Shu W, Giri DD, Viale A, Olshen AB, Gerald WL, Massague J: **Genes that mediate breast cancer metastasis to lung**. *Nature* 2005, **436**(7050):518-524.
6. Sabatier R, Finetti P, Adelaide J, Guille A, Borg JP, Chaffanet M, Lane L, Birnbaum D, Bertucci F: **Down-regulation of ECRG4, a candidate tumor suppressor gene, in human breast cancer**. *PLoS One* 2011, **6**(11):e27656.
7. Sircoulomb F, Bekhouche I, Finetti P, Adelaide J, Ben Hamida A, Bonansea J, Raynaud S, Innocenti C, Charafe-Jauffret E, Tarpin C *et al*: **Genome profiling of ERBB2-amplified breast cancers**. *BMC Cancer* 2010, **10**:539.
8. Ivshina AV, George J, Senko O, Mow B, Putti TC, Smeds J, Lindahl T, Pawitan Y, Hall P, Nordgren H *et al*: **Genetic reclassification of histologic grade delineates new clinical subtypes of breast cancer**. *Cancer Res* 2006, **66**(21):10292-10301.
9. Pawitan Y, Bjohle J, Amler L, Borg AL, Egyhazi S, Hall P, Han X, Holmberg L, Huang F, Klaar S *et al*: **Gene expression profiling spares early breast cancer patients from adjuvant therapy: derived and validated in two population-based cohorts**. *Breast Cancer Res* 2005, **7**(6):R953-964.
10. Silver DP, Richardson AL, Eklund AC, Wang ZC, Szallasi Z, Li Q, Juul N, Leong CO, Calogrias D, Buraimoh A *et al*: **Efficacy of neoadjuvant Cisplatin in triple-negative breast cancer**. *J Clin Oncol* 2010, **28**(7):1145-1153.
11. Dedeurwaerder S, Desmedt C, Calonne E, Singhal SK, Haibe-Kains B, Defrance M, Michiels S, Volkmar M, Deplus R, Luciani J *et al*: **DNA methylation profiling reveals a predominant immune component in breast cancers**. *EMBO Mol Med* 2011, **3**(12):726-741.
12. Lu X, Wang ZC, Iglehart JD, Zhang X, Richardson AL: **Predicting features of breast cancer with gene expression patterns**. *Breast Cancer Res Treat* 2008, **108**(2):191-201.
13. **The International Genomics Consortium (IGC). The expO project (Expression Project for Oncology)** [www.intgen.org]

14. Schmidt M, Bohm D, von Torne C, Steiner E, Puhl A, Pilch H, Lehr HA, Hengstler JG, Kolbl H, Gehrmann M: **The humoral immune system has a key prognostic impact in node-negative breast cancer**. *Cancer Res* 2008, **68**(13):5405-5413.
15. Gluck S, Ross JS, Royce M, McKenna EF, Jr., Perou CM, Avisar E, Wu L: **TP53 genomics predict higher clinical and pathologic tumor response in operable early-stage breast cancer treated with docetaxel-capecitabine +/- trastuzumab**. *Breast Cancer Res Treat* 2012, **132**(3):781-791.
16. Richardson AL, Wang ZC, De Nicolo A, Lu X, Brown M, Miron A, Liao X, Iglehart JD, Livingston DM, Ganesan S: **X chromosomal abnormalities in basal-like human breast cancer**. *Cancer Cell* 2006, **9**(2):121-132.
